# Directly Converted Human Fibroblasts Mature to Neurons and Show Long-Term Survival in Adult Rodent Hippocampus

**DOI:** 10.1155/2017/5718608

**Published:** 2017-11-26

**Authors:** Natalia Avaliani, Ulrich Pfisterer, Andreas Heuer, Malin Parmar, Merab Kokaia, My Andersson

**Affiliations:** ^1^Epilepsy Centre, Lund University Hospital, Lund, Sweden; ^2^Developmental Neurobiology, Lund University, Lund, Sweden; ^3^Molecular Neuromodulation, Lund University, Lund, Sweden

## Abstract

Direct conversion of human somatic cells to induced neurons (iNs), using lineage-specific transcription factors has opened new opportunities for cell therapy in a number of neurological diseases, including epilepsy. In most severe cases of epilepsy, seizures often originate in the hippocampus, where populations of inhibitory interneurons degenerate. Thus, iNs could be of potential use to replace these lost interneurons. It is not known, however, if iNs survive and maintain functional neuronal properties for prolonged time periods in *in vivo*. We transplanted human fibroblast-derived iNs into the adult rat hippocampus and observed a progressive morphological differentiation, with more developed dendritic arborisation at six months as compared to one month. This was accompanied by mature electrophysiological properties and fast high amplitude action potentials at six months after transplantation. This proof-of-principle study suggests that human iNs can be developed as a candidate source for cell replacement therapy in temporal lobe epilepsy.

## 1. Introduction

During the past decade, the discovery of reprograming somatic cells by endogenous expression of a few transcription factors into a pluripotent stage [1, 2], or somatic cells of another phenotype [3–6], has created new opportunities for disease modelling and cell replacement therapies. Patient-specific induced pluripotent stem cells (iPS) have already been proven useful for modelling a wide range of neurological diseases [7–9]. It has been shown that they can be generated from multiple somatic cell sources [10], which makes them an attractive source for the development of cell therapy. There is still, however, a substantial risk of tumorigenesis associated with the pluripotent stage [11, 12]. An alternative for generating patient-specific neurons of somatic origin is directly converted induced neurons (iNs). Vierbuchen et al. demonstrated that by using three neuronal lineage-inducing transcription factors, Brn2, AsclI, and Myt1b (BAM), mesodermal mouse fibroblasts could efficiently be converted into neurons, bypassing the pluripotent state, thereby decreasing the probability of uncontrolled proliferation and teratoma formation after transplantation [13].

Several groups have shown that with the BAM approach it is possible to derive iNs from different cell sources and, through varied combinations of factors, direct the cells into specific neuronal subtype, for example, dopaminergic [14], cholinergic [15], or spinal motor neurons [16]. Functional iNs with mature electrophysiological properties have already been obtained from both rodent and human somatic cells, ranging from embryonic and postnatal to adult fibroblast cell populations [17]. It has also been shown *in vitro* that this process is much faster than deriving mature, functional neurons from iPS cells [18, 19] and that human iNs survive and express neuronal markers after four weeks *in vivo* and can persist for over six months after transplantation into the striatum [20, 21]. It is not known, however, if these cells will survive and mature after transplantation into the adult hippocampus and if surviving cells are stably reprogrammed and maintain neuronal characteristics over time *in vivo*. This knowledge is of particular importance for using iNs as a potential cell replacement therapy against one of the most severe and pharmacoresistant forms of epilepsy, where seizures often originate in the temporal lobe, in particular, in the hippocampus, and where certain interneuron populations degenerate [22–24].

To this end, we transplanted iNs derived from foetal human lung fibroblast into the hippocampus of adult rats. The human iNs showed mature morphological and electrophysiological properties at six months after transplantation, indicating the long-term survival and functionality, and thereby compatibility with the cell replacement therapeutic strategy in temporal lobe epilepsy.

## 2. Materials and Methods

### 2.1. Animals

Male immunodeficient nude rats (NIH Nude rat, Charles River), weighing 250 g, were housed under a 12/12-hour light cycle with ad libitum access to water and food in individually ventilated cages, 24 animals assigned to hippocampus transplantation and 4 animals for striatal transplantation. The Lund Ethical Committee for Experimental Animals approved all experimental procedures, and all experiments were performed in accordance with relevant guidelines and regulations.

### 2.2. Adenoassociated Viral Vector Injections

For selective activation of host cells posttransplant, the blue light-activated channelrhodopsin-2 (ChR2) was expressed in a nude rat hippocampus with the adenoassociated viral vector, AAV5-hSyn-hChR2(H134R)pEYFP (Addgene, plasmid number 26973). The vector was stereotactically injected bilaterally in isoflurane-anaesthetized rats. A total amount of 3 *μ*l virus suspension (titer of 2^∗^10^12^ particles/ml) was injected through a glass capillary at 0.1 *μ*l/min rate in the following coordinates: anterior-posterior (AP) −6.2 mm; medial-lateral (ML) ±5.2 mm; and dorsal-ventral (DV) −6.0, −4.8, and −3.6 mm, 1.5 *μ*l at each location in a DV plane. The reference points used were the bregma for AP, midline for ML, and dura for DV. The glass capillary was left in each DV point for 5 minutes after injection to prevent backflow of the viral particles through the injection track.

### 2.3. Generation and Transplantation of iN Cells

Foetal human lung fibroblast cells (HFL1; ATCC-CCL-153, ATCC®, USA), obtained from the American Type Culture Collection, were cultured in DMEM medium (Life Technologies), supplemented with 10% of FBS (Sigma-Aldrich), 2 mM L-glutamine, and 1% of penicillin/streptomycin (both from Invitrogen). The fibroblasts were transduced with doxycycline-regulated lentiviruses (LVs), expressing mouse cDNAs for BAM, plus the TET-ON transactivator (FuW.rtTA-SM2, Addgene), cotransduced during conversion, as previously described [20]. The LVs were used at a multiplicity of infection (MOI) of 5 for BAM and 10 for mrtTA-FUW. In addition, RFP was expressed in converted cells, using a retroviral construct under a CAG promoter, with a MOI of 5. Doxycycline (2 *μ*g/mL) was added to the culture medium 5 days after transduction, and 1 mg/ml was given in drinking water to the cell-transplanted rats one week before and 12 weeks postgrafting, continuing the conversion *in vivo*. Two days after transgene activation, MEF medium was replaced by neural differentiation medium (N2B27; Stem Cells), supplemented with small molecules (SMs) with the following concentrations: 2 *μ*M CHIR99021 (Axon), 10 *μ*M SB431542, 100 ng/ml Noggin (R&D Systems), and 0.5 *μ*M LDN-193189 (Axon), and growth factors at the following concentrations: 2 ng/ml LM4A22 (R&D Systems), 2 ng/ml GDNF, 10 ng/ml NT3 (R&D Systems), and 0.5 mM db-cAMP (Sigma-Aldrich). Three-quarters of the medium in the wells were changed every 2nd–3rd day, until the day of transplantation.

For intrahippocampal and intrastriatal transplantation, cells were resuspended in N2B27 medium to reach a concentration of 100,000 cells/*μ*l and injected stereotactically. Striatal coordinates were the following: AP +0.5 mm, ML −3.0 mm, and DV −5.0 and −4.0 mm, 2 *μ*l at each DV coordinate. Both hippocampi received cell transplants in the same coordinates as the virus injection, 3 *μ*l in total per hippocampus (1 *μ*l at each DV coordinate). To ensure extracellular virus clearance, the transplantation was performed one week after AAV virus injection [25].

### 2.4. Acute Hippocampal Slice Preparation

Acute hippocampal slices were prepared from iN cell-transplanted nude rats, as previously described [26] at one and six months after grafting. Briefly, for each recording day, one rat was anaesthetized and decapitated, the brain removed from the skull and rapidly immersed in ice-cold cutting solution containing (in mM) the following: sucrose 75, NaCl 67, NaHCO3 26, glucose 25, KCl 2.5, NaH2PO4 1.25, CaCl2 0.5, and MgCl2 7 (pH 7.4, osmolarity 300 mOsm). The hemispheres were divided and placed on a cutting stage as described elsewhere [27]. Transverse slices of 300 *μ*m thickness comprising the hippocampus as well as the entorhinal cortex were cut on a vibratome (VT1200S, Leica Microsystems) and immediately transferred to an incubation chamber filled with artificial cerebrospinal fluid (aCSF) containing (in mM) the following: NaCl 119, KCl 2.5, MgSO_4_ 1.3, NaHCO_3_ 26.2, NaH_2_PO_4_ 1, glucose 11, and CaCl_2_ 2.5 (300 mOsm, pH 7.4), oxygenated with carbogen and maintained at 34°C, where the slices were allowed to rest for one hour before the experiments started.

### 2.5. Electrophysiology

Individual slices were placed in the recording chamber, continuously perfused at a rate of 3 ml/min with carbogenated aCSF and kept at a temperature of 32–34°C.

RFP-expressing cells in the slices were identified using a wide-band excitation filter and visualized for whole-cell patch clamp recordings using infrared differential interference contrast video microscopy (BX50WI; Olympus). The recording glass pipette was backfilled with solution containing (in mM) the following: K-gluconate 122.5, KCl 17.5, NaCl 8, KOH-HEPES 10, KOH-EGTA 0.2, MgATP 2, and Na3GTP 0.3 (295 mOsm, pH 7.2; all from Sigma-Aldrich). Average pipette tip resistance was between 3–5 MΩ. Pipette current was corrected online before gigaseal formation while fast capacitive currents were compensated for during cell-attached configuration. Only experiments with series resistance values of less than 20 MΩ were selected for the analysis. Biocytin was included in the pipette solution at 0.5–1 mg/ml to retrospectively identify recorded cells. All recordings were done using a HEKA EPC10 amplifier (HEKA Elektronik, Germany) and sampled at 10 kHz.

Resting membrane potential (RMP) was recorded in current-clamp mode at 0 pA immediately after establishing whole-cell configuration. Series resistance and input resistance (Ri) were calculated from a 5 mV pulse and monitored throughout the experiment. Action potential (AP) threshold was determined by 500 ms square current step injections at RMP, with 20 pA increments. Ramp injection of 1 s current was used to determine action potential threshold in addition to step depolarization. AP amplitude was measured from threshold to peak, and duration was measured as the width at the threshold. Spontaneous postsynaptic currents (sPSCs) were measured at −70 mV. Voltage steps were delivered in 10 mV (200 ms) increments in voltage-clamp mode at a holding potential of −70 mV.

### 2.6. Optogenetic Stimulation

For optogenetic depolarization of ChR2-expressing cells, blue light was applied at 460 nm wavelength with a LED light source (Prizmatix, Modi'in Illit, Israel) and delivered through a water immersion 40x microscope objective. Stimulation of ChR2-expressing cells was done either by continuous application of blue light for 10s or by 1 ms paired pulses separated by 100 ms intervals.

### 2.7. Immunohistochemistry and Imaging

Rats with iN grafts in the striatum were perfused with ice-cold 4% paraformaldehyde solution in phosphate buffer (PB) one month posttransplantation, and brains were cut as series of 30 *μ*m coronal slices on a microtome. All slice preparations were fixed in 4% paraformaldehyde solution in PB buffer (for 12 to 24 h) after patch-clamp recordings, then rinsed with Kalium-PBS (KPBS), and stored in Walter's antifreeze medium (ethylene glycol and glycerol in PB) at −20°C. For immunohistochemistry, slices were washed three times in KPBS and incubated in 1% Triton X-100-KPBS (tKPBS)/10% normal goat 5 serum (NGS) for one hour followed by 1% tKPBS/10% NGS with primary antibody. Primary antibodies were applied overnight at room temperature in concentrations of 1 : 500 for rabbit anti-RFP (Abcam) and mouse anti-TE7 (Sigma-Aldrich) while 1 : 100 was used for mouse anti-hNCAM (Abcam). Slices were rinsed three times in KPBS solution and incubated for 2 hours in RT with secondary antibodies in the concentration of 1 : 400 for Cy3 anti-rabbit and Cy5 anti-mouse, while 1 : 300 was used for Cy5 streptavidin (all from Invitrogen). The slices are then mounted with DABCO (Sigma-Aldrich). For DAB staining, brain sections were incubated with biotinylated secondary antibody, followed by streptavidin-horseradish peroxidase (Vector Laboratories) and then exposed to DAB (0.5 mg/ml, Sigma-Aldrich) together with 0.01% hydrogen peroxide for 5 min.

Images were acquired either by confocal (Inverted Nikon Eclipse Ti microscope Csi) or by epifluorescence microscopy (Olympus BX61). Morphological analysis was performed in ImageJ using the free plugin NeuronJ for the quantification of soma circumference and distance to first branching [28].

### 2.8. Statistical Analysis

Statistical comparisons were made using ANOVA followed by Tukey's post hoc test and Student's *t*-test.

## 3. Results

### 3.1. Directly Converted Neurons Survive Six Months after Transplantation

**Figure 1:**
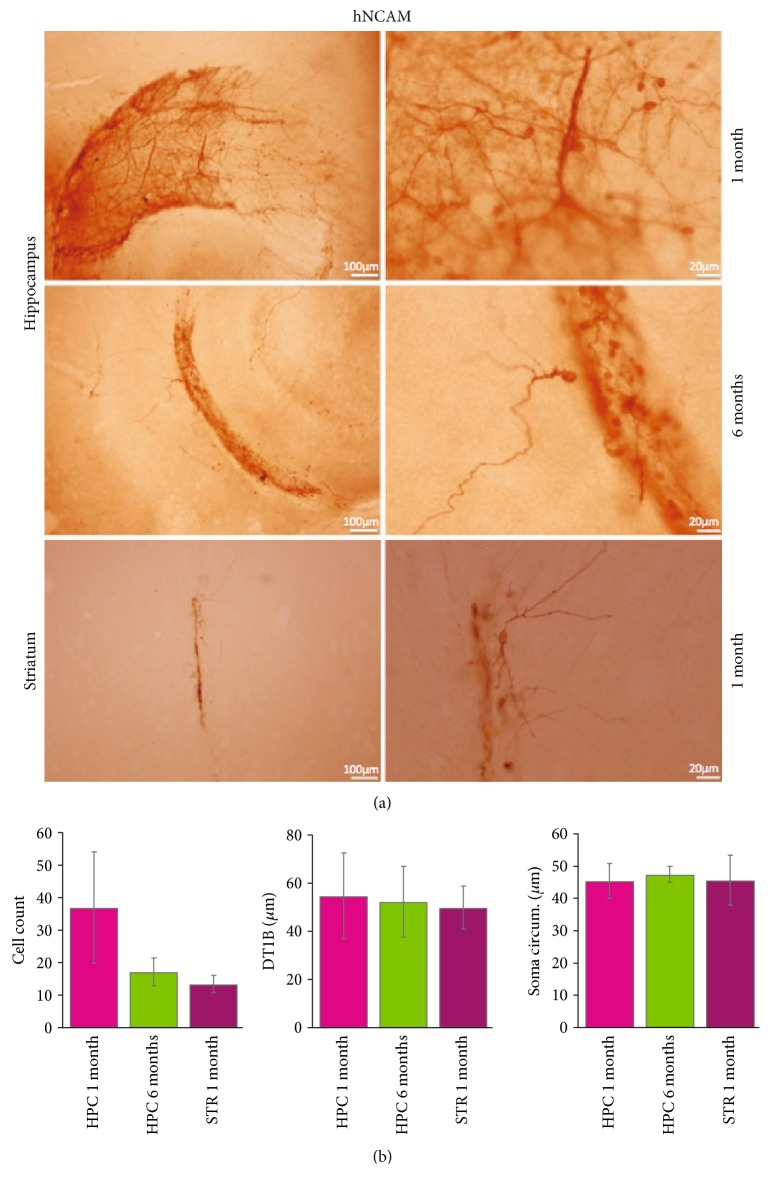
Human lung fibroblasts directly converted to human neurons survive for six months transplanted into the adult rat hippocampus. (a) Transplanted human lung fibroblasts, directly converted with the BAM factors, express human neuronal cell adhesion molecule (hNCAM) already at one month postgrafting in adult rat hippocampus (top third) and striatum (lower third). The converted cells survive and keep their neuronal appearance and hNCAM expression for six months in the hippocampus (middle third). (b) No difference between regions or time points was observed when studying the gross neuronal morphology observed in hNCAM-labelled cells either in cell number, distance to first branching point (DT1BP), or soma circumference (soma circum.).

In order to investigate if human iN, transplanted to the adult hippocampus, survive over time, foetal human lung fibroblasts were transduced with BAM, treated with small molecules (SM) [20] and transplanted to the hippocampus of immunocompromised rats. Animals were given doxycycline in the drinking water, continuing activation of conversion after transplantation. In a separate experiment, we performed cell transplantation to the striatum in a subset of animals as a positive control to compare survival and morphological properties of iNs grafted into two different brain structures. In both cases, transplanted cells showed substantial survival at one month and formed neuron-rich grafts, identified by human-specific neural cell adhesion molecule (hNCAM) immunostaining (Figure 1(a)). Grafted iNs showed no difference in morphological characteristics transplanted to the striatum or hippocampus (Figure 1(b)), as quantified by cell number (HPC 1 month, 37.0 ± 1, *n* = 3; HPC 6 months, 17.2 ± 4.2, *n* = 5; and striatum 1 month, 13.4 ± 2.7, *n* = 5), soma circumference (HPC 1 month, 45.4 ± 5.4, *n* = 16; HPC 6 months, 47.5 ± 2.6, *n* = 6; and striatum 1 month, 45.7 ± 7.7, *n* = 6), and distance to first branching (DT1B, HPC 1 month, 54.7 ± 17.7, *n* = 11; HPC 6 months, 52.3 ± 14.8, *n* = 5; and striatum 1 month, 49.9 ± 9.0, *n* = 5), with no statistical difference between the groups. Similar values were also observed in the hippocampus six months after transplantation (Figure 1(b)) suggesting that at one month the cells have developed their gross morphological characteristics, which is maintained throughout the period studied.

**Figure 2:**
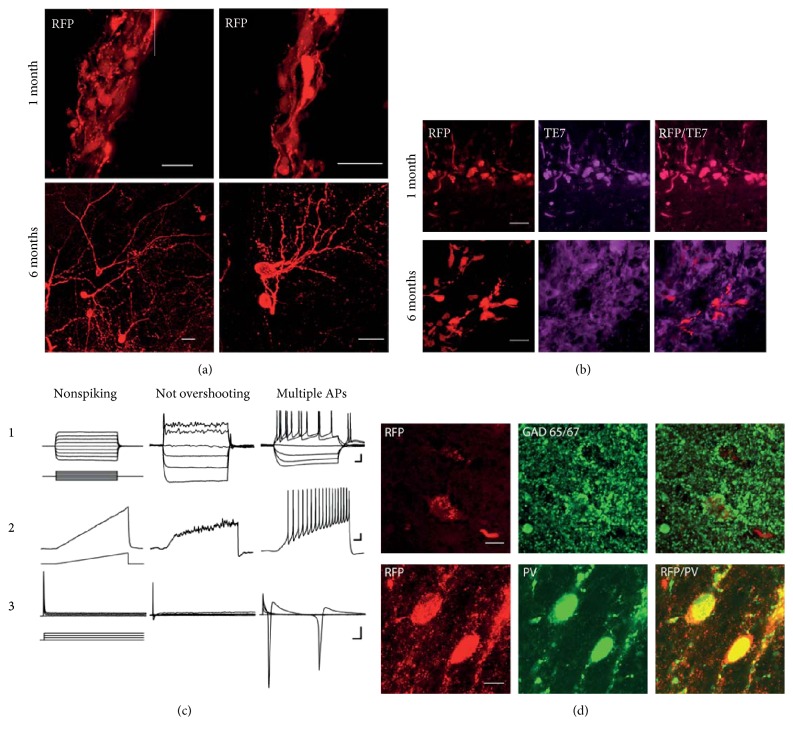
(a) Converted human iNs display complex branching after six months *in vivo*; converted RFP-expressing human iNs keep their neuronal morphology and extend their branching from one to six months after transplantation. Scale bars, 20 *μ*m. (b) Cells expressing the fibroblast markers, TE7 and RFP, are found both in the hippocampus and in the striatum at one month postgrafting. At 6 months, unconverted cells expressing TE7 can still be found. RFP-expressing cells, however, do not express TE7 at this time point. Scale bars: 20 *μ*m. (c) Three electrophysiological profiles of iN cells are found six months after transplant. Representative traces of whole-cell recordings for the cells that do not fire any APs (nonspiking), the cells that fire one nonovershooting AP (not overshooting), and the cells that fire multiple APs (multiple APs). ((c), 1) Membrane potential changes induced by current injection steps with 20 pA increments at RMP. ((c), 2) Ramp depolarization from 0 to 50 pA. ((c), 3) 10 mV depolarization voltage steps from −70 mV induce small inward sodium current in the cells with single not overshooting AP and big inward sodium and outward potassium currents in the mature cells with multiple APs. Scale bars: (a) 20 mV/50 ms; (b) 10 mV/100 ms; and (c) 250 pA/5 ms. (d) A proportion of iN cells matures into GABAergic neurons expressing interneuron markers. Immunostaining for GABA and RFP revealed colocalisation in induced neurons, and staining for interneuron-specific marker parvalbumin (PV) showed presence of PV interneurons amongst directly converted neurons.

By taking advantage of RFP expression, we could follow the extensive arborisation and detailed morphology of the transplanted cells (Figure 2). As doxycycline was removed after three months, the conversion appeared stable without a continuous supply of the exogenous conversion factors and the induced neurons kept and developed their neuronal morphology, seen as increased length of processes and increased neurite branching (Figure 2(a)). Staining for the fibroblast marker, TE7 [29], showed that a portion of the transplanted cells remained unconverted in the hippocampus at both one and six months postgrafting (Figure 2(b)). We could not find, however, any RFP-positive cells coexpressing TE7 after six months (Figure 2(b)).

#### 3.2. Converted Neurons Show Functional Neuronal Properties

**Figure 3:**
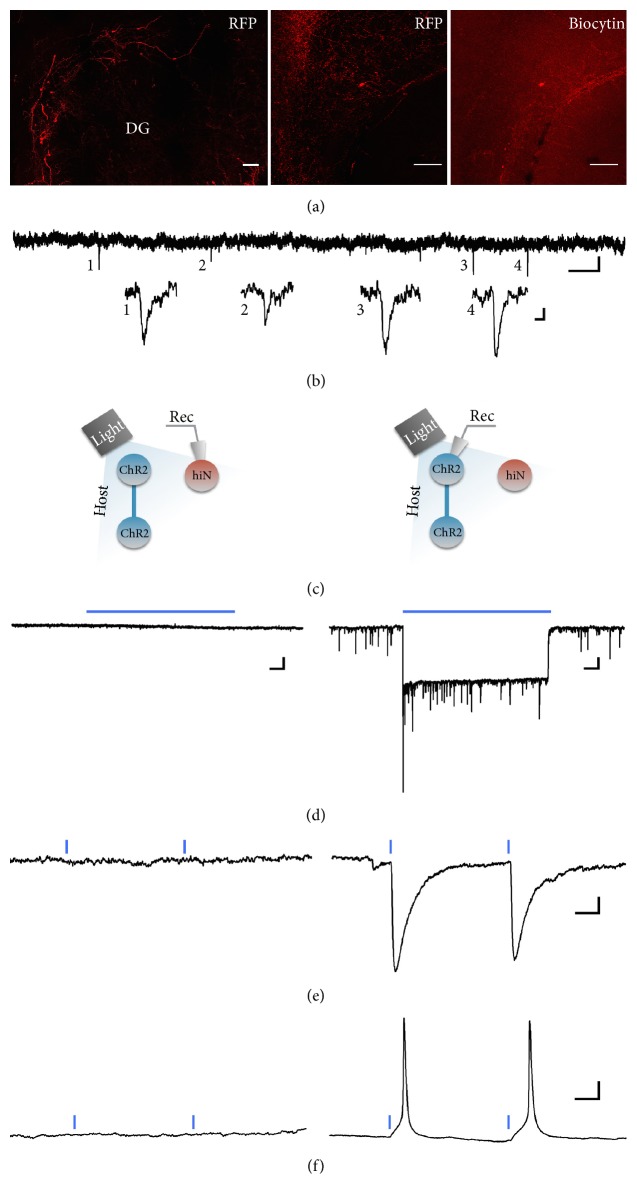
iN cells with mature neuronal profile exhibit sparse synaptic integration. (a) Immunostaining of hippocampal slice with RFP-labelled iN grafts (on the left). Middle image shows higher magnification with the recorded iN cell, also identified by biocytin staining (on the right). (b) Continuous voltage clamp recording of grafted iN cell at −70 mV, showing sparse PSCs, with magnified insets (numbered 1 to 4). (c) Schematic explanation of optogenetic activation of ChR2-expressing host tissue, while recording from a grafted iN (left), or a ChR2-expressing host cell (right), for comparison; traces shown in (d–f). Blue light activation of ChR2-expressing host tissue does not result in synaptic response in the transplanted iN cells (left), neither in voltage-clamp mode, while applying blue light continuously for 10s (d) or as 1 ms paired pulses (e), nor in current-clamp mode (f), while there is a clear strong response to blue light in the host ChR2-expressing cells (d, e, right), with light-induced APs in current-clamp mode (f). Blue lines indicate the place and time of light application. Note the difference in scale in (d) traces between iN and host cells. Scale bars: (a) 100 *μ*m; (b) 5 pA/1 s; (c) insets 2 pA/10 ms; (d) 50 pA/1 s (left) and 20 pA/1 s (right); (e) 20 pA/20 ms; and (f) 10 mV/10 ms.

After morphological characterization, we next evaluated functional neuronal properties of the transplanted cells at six months after transplantation. The conversion of human fibroblasts to neurons is a process that requires rearrangements of the cell membrane, such as the insertion of various ion channels during the neural conversion process. We recorded electrophysiological properties from RFP-expressing cells with neuronal morphology at six months posttransplantation. The average input resistance of the iNs was 867.9 ± 172.2 *Ω*, *n* = 7 with a RMP of −63.6 ± 6.1 mV. The passive membrane properties, such as RMP, were in line with a mature neuronal phenotype. The ability to fire action potentials (APs), however, varied amongst these neurons, with three major neuronal profiles identified (Figure 2(c)): (1) nonfiring iN cells that were not able to generate action potentials (*n* = 3); (2) iN cells that fired a single, broad AP with ~6 ms duration (*n* = 2); and (3) iN cells able to fire fast repetitive APs (*n* = 2). The electrophysiological parameters of the iNs in the third group were similar to those of mature human neurons [26], with AP amplitudes of 65 and 80 mV, AP durations of 3 and 2.4 ms, and AP thresholds of −43 and −40 mV. The neurons in the third group also displayed delayed-rectifier outward potassium currents after the inward sodium currents (Figure 2(c), 3) and exhibited spontaneous postsynaptic currents (SPSCs, exemplified in Figure 3(b)), indicating the existence of afferent synapses. Immunostaining for GABA and RFP revealed colocalisation in induced neurons suggesting that these neurons are capable of forming inhibitory synapses (Figure 3(d)). Indeed, staining for interneuron-specific marker parvalbumin (PV) confirmed that a proportion of converted neurons becomes PV interneurons.

To differentiate between synaptic inputs from graft and host neurons, ChR2 was expressed in the host hippocampus one week before cell transplantation, using the pan-neural human synapsin promoter (hSyn), which enabled host neurons to be readily activated by blue light, delivered through the 40x objective (schematically illustrated in Figure 3(c)). We were not able, however, to detect any synaptic currents from the recorded iNs in response to host neuron light activation (*n* = 7, recorded from 6 animals, Figures 3(d)–3(f), left), suggesting that the majority of the spontaneous postsynaptic events observed in these cells were generated by synaptic connections between grafted iNs. The number of recorded cells was too low, however, for any conclusions to be drawn about potential integration efficacy of transplanted cells in the host network.

## 4. Discussion

We show, for the first time, the long-term survival of human iNs, transplanted into the adult rodent hippocampus, with functional neuronal properties.

We could not detect any differences in gross morphology measured as soma size, distance to first branching, or cell number (Figure 1) between transplants in the hippocampus and striatum, suggesting that the initial conversion is largely unaffected by the surrounding environment. Directly converted cells need time to change phenotype, a process that requires the development of neuronal processes, rearrangement of the cell membrane, and expression of various ion channels. This process is asynchronous in the cell population, and it has been shown to take several weeks *in vitro* for human iNs to display mature neuronal properties [20, 21, 30]. The neuronal morphology at the six-month time point displayed extensive branching (Figure 2(a)), with no change in soma size compared to the first month (Figure 1). When recording intrinsic membrane properties with whole-cell patch clamp after six months *in vivo*, the population of cells is still heterogeneous in terms of maturation (Figure 2(b).), indicating that it is the initial rate of conversion that decides phenotype not time after transplantation. This is in line with findings that subtypes of cells with different levels of conversion can be found up to 28 days during human iN conversion *in vitro* [30]. This is further supported by the presence of TE7-expressing cells at both one and six months after transplantation. We could not find, however, RFP-expressing neurons coexpressing TE7 after six months (Figure 2(b)).

The cells that fired action potentials also displayed postsynaptic currents (Figure 3), suggesting that converted cells that reach basic neuronal properties also have the ability to integrate synaptically. A proportion of converted neurons was positive for GABA and the specific interneuron marker PV, suggesting that these neurons have the capacity to form inhibitory GABAergic synapses (Figure 2(d)).

We were unable to elicit postsynaptic current by optogenetic activation of host cells, suggesting that the synaptic currents recorded were synapses made between transplanted cells or non-ChR2-expressing host cells. As the number of recorded cells was low, however, we did not have the opportunity to fully evaluate the efficacy of synaptic integration of iNs to host neurons.

## 5. Conclusions

This study shows the potential for iN cells to function as a strong candidate for cell replacement therapy in neurological diseases affecting the adult CNS and the hippocampus in particular. A possible target is temporal lobe epilepsy, which displays loss of specific subpopulations of interneurons in the hippocampus, and cell replacement therapy of lost interneurons [22, 23] has resulted in marked reduction in seizures in animal models of TLE [31, 32]. Further studies, following conversion, survival, and integration rate of transplanted cells, are needed before these cells can be considered for cell replacement therapy.
